# Anti-quorum Sensing and Protective Efficacies of Naringin Against *Aeromonas hydrophila* Infection in *Danio rerio*

**DOI:** 10.3389/fmicb.2020.600622

**Published:** 2020-12-03

**Authors:** Ramanathan Srinivasan, Kannan Rama Devi, Sivasubramanian Santhakumari, Arunachalam Kannappan, Xiaomeng Chen, Arumugam Veera Ravi, Xiangmin Lin

**Affiliations:** ^1^Fujian Provincial Key Laboratory of Agroecological Processing and Safety Monitoring, School of Life Sciences, Fujian Agriculture and Forestry University, Fuzhou, China; ^2^Key Laboratory of Crop Ecology and Molecular Physiology, Fujian Agriculture and Forestry University, Fujian Province University, Fuzhou, China; ^3^Department of Biotechnology, Alagappa University, Karaikudi, India; ^4^Department of Biochemistry and Molecular Biology, School of Life Sciences, Pondicherry University, Pondicherry, India; ^5^Department of Food Science and Technology, School of Agriculture and Biology, Shanghai Jiao Tong University, Shanghai, China; ^6^Key Laboratory of Marine Biotechnology of Fujian Province, Institute of Oceanology, Fujian Agriculture and Forestry University, Fuzhou, China

**Keywords:** *Aeromonas hydrophila*, biofilm, naringin, quorum sensing, virulence factors, zebrafish

## Abstract

It is now well known that the quorum sensing (QS) mechanism coordinates the production of several virulence factors and biofilm formation in most pathogenic microorganisms. *Aeromonas hydrophila* is a prime pathogen responsible for frequent outbreaks in aquaculture settings. Recent studies have also continuously reported that *A. hydrophila* regulates virulence factor production and biofilm formation through the QS system. In addition to the presence of antibiotic resistance genes, biofilm-mediated antibiotic resistance increases the severity of *A. hydrophila* infections. To control the bacterial pathogenesis and subsequent infections, targeting the QS mechanism has become one of the best alternative methods. Though very few compounds were identified as QS inhibitors against *A. hydrophila*, to date, the screening and identification of new and effective natural QS inhibitors is a dire necessity to control the infectious *A. hydrophila*. The present study endorses naringin (NA) as an anti-QS and anti-infective agent against *A. hydrophila*. Initially, the NA showed a concentration-dependent biofilm reduction against *A. hydrophila*. Furthermore, the results of microscopic analyses and quantitative virulence assays displayed the promise of NA as a potential anti-QS agent. Subsequently, the downregulation of *ahh1*, *aerA*, *lip* and *ahyB* validate the interference of NA in virulence gene expression. Furthermore, the *in vivo* assays were carried out in zebrafish model system to evaluate the anti-infective potential of NA. The outcome of the immersion challenge assay showed that the recovery rate of the zebrafish has substantially increased upon treatment with NA. Furthermore, the quantification of the bacterial load upon NA treatment showed a decreased level of bacterial counts in zebrafish when compared to the untreated control. Moreover, the NA treatment averts the pathogen-induced histoarchitecture damages in vital organs of zebrafish, compared to their respective controls. The current study has thus analyzed the anti-QS and anti-infective capabilities of NA and could be employed to formulate effective treatment measures against *A. hydrophila* infections.

## Introduction

The genus *Aeromonas* is a challenging group of microorganisms to treat for physicians and microbiologists due to their notorious role in causing several infectious diseases (Janda and Abbott, 2010). It engages in a variety of human illnesses such as gastroenteritis, hemolytic uremic syndrome, bacteremia, septicemia, meningitis, peritonitis, wound infections, and respiratory tract and ocular infections (Li et al., 2020). This study mainly focuses on *Aeromonas hydrophila*, which is an important species in the genus *Aeromonas* that has posed a potential health threat to fishes and humans especially (Rama Devi et al., 2016). *A. hydrophila* has been recognized as an opportunistic pathogen causing infections in immunocompromised patients, and it was recently found to cause foodborne illness in healthy individuals (Daskalov, 2006). It produces a wide range of extracellular enzymes, which are thought to be key players in causing infections in immunocompromised individuals.

Biofilm is the favorite mode of growth for most bacteria. Biofilm is an assemblage of microbial cells on a surface with an enclosed polysaccharide matrix (Donlan, 2002). This biofilm formation is intimately related to a population-dependent gene expression system known as quorum sensing (QS), which utilizes small self-produced chemicals called autoinducers (AIs) (Fuqua et al., 2001; Srinivasan et al., 2016). Generally, *A. hydrophila* is known to produce three different types of AIs, such as N-butanoyl-homoserine lactone (C4-HSL), N-hexanoyl-homoserine lactone (C6-HSL), and AI-2, for their communication (Kozlova et al., 2008). In addition to biofilm formation, *A. hydrophila* secretes a wide array of virulence factors, such as aerolysin, cytotoxic enterotoxins, elastase, hemolysins, lipases, proteases, and an S layer under the control of QS system (Rama Devi et al., 2016). These virulence factors of *A. hydrophila* have the symptomatic potential to cause severe diseases in fishes and humans (Murray et al., 1988; Chopra and Houston, 1999; Cascón et al., 2000; Singh et al., 2009; Khajanchi et al., 2010; Shak et al., 2011).

The hemolysin production in *A. hydrophila* is controlled by two-component hemolytic systems like hemolysin (*ahh1*) and aerolysin (*aerA*) (Wang et al., 2003). These extracellular hemolysin enzymes invade the host cell membrane’s lipid bilayer and lead to the leakage of cytoplasmic content. Furthermore, the extracellular hydrolytic enzyme elastase (*ahyB*) is an important virulence trait in *A. hydrophila*, which causes cell damage when associated with aerolysin (Song et al., 2004). Another extracellular hydrolytic enzyme in *A. hydrophila* is lipase, which affects the host’s immune system functions by generating free fatty acids through lipolytic activity (Eftimiadi et al., 1987).

The use of antibiotics is the gold-standard treatment strategy to contain the human illness caused by *A. hydrophila* (Morris and Horneman, 2013). However, the current rise in antibiotic resistance among aeromonads has urged an interest in developing a promising alternative treatment strategy (Li et al., 2019). Since QS plays a key role in the pathogenesis and survival of *A. hydrophila*, targeting QS has become an alternate approach to contain *A. hydrophila* infection. Naringin (NA) [4′,5,7-trihydroxyflavanone-7-β-D-α-L-rhamnosyl(1→2)-β-D-glucoside] is a flavanone glycoside predominantly found in citrus fruits and particularly in grapefruit and sour orange (Peterson et al., 2006a,b). It is a prime bitter component in grapefruit and its level varies with different cultivars. In recent years, flavonoids from citrus fruits have gained much importance in drug development research because of their proven medicinal values (Rawson et al., 2014; Salvamani et al., 2014). The current study aimed to explore the anti-QS potential of NA against *A. hydrophila* and its efficacy in rescuing *Danio rerio* (animal model) from *A. hydrophila* infection.

## Materials and Methods

### Bacterial Strain, Growth Condition, and Media

*A. hydrophila* MTCC 1739 was obtained from the Microbial Type Culture Collection & Gene Bank, Institute of Microbial Technology (IMTECH), Chandigarh, India. The test strain was routinely grown at 30°C in Luria Bertani (LB) broth with rotary shaking at 130 rpm. The OD of the test bacterial pathogen was adjusted to 0.4 at 600 nm (UV-visible spectrophotometer; Shimadzu, UV-2450, Japan) from the overnight culture (1 × 10^8^ CFU/ml) and used as the standard cell suspension for all the *in vitro* assays.

### Determination of Minimum Biofilm Inhibitory Concentration (MBIC)

The MBIC of NA was tested against *A. hydrophila* in a 24-well microtiter plate (MTP). NA at doubled dilution concentrations (93.75, 187.5, 375, 750, and 1,500 μg/ml) was added to the MTP wells containing 1 ml of LB broth, and 1% of *A. hydrophila* standard cell suspension was used as inoculum. The MTP was incubated for 24 h at 30°C in a static condition. Control was maintained without the addition of NA. After incubation, the planktonic cells and the spend media were discarded from the control and NA treated wells. Then, the wells were washed with sterile distilled water and allowed to air dry. Further, the wells were stained with 0.4% crystal violet (CV) (w/v) solution at room temperature for 5 min. The unbound CV was washed with sterile distilled water and air dried. A total of 1 ml of 20% glacial acetic acid solution was added to all wells to extract the biofilm bound CV and read spectrophotometrically at 570 nm to quantify the biofilm biomass (Kannappan et al., 2019a). The percentage of biofilm inhibition was calculated by using the following formula:

Percentage⁢of⁢inhibition=[(controlOD-570nmtreatedOD)570nm/controlOD]570⁢nm×100

MBIC was determined as the lowest concentration of NA that holds the maximum percentage of biofilm inhibition without any reduction in bacterial growth.

### QS Inhibition Assay in *A. hydrophila*

*A. hydrophila* was grown at 30°C for 24 h in the absence and presence of NA (93.75–750 μg/ml). After incubation, the cultures were harvested, and cell-free culture supernatants (CFCS) were collected and then filter sterilized using a 0.22 μm membrane filter (Millipore Corp., United States) and used for further studies.

#### β-Hemolysin Quantification Assay

The extracellular hemolysin production in *A. hydrophila* was quantified using the method described by Scheffer et al. (1988). Briefly, 100 μl of both NA treated and untreated *A. hydrophila* CFCS were added with 900 μl of phosphate buffer saline (PBS; pH 7.4) containing 2% sheep erythrocytes. The mixture was left undisturbed for 20 min in ice. The mixture was then centrifuged, and the absorbance for the released hemoglobin in the supernatant was measured at 530 nm.

#### Lipase Assay

The lipase production was quantitatively evaluated by *p*-nitrophenyl palmitate as a substrate. A total of 100 μl of NA treated and untreated CFCS were added to 900 μl of substrate mixture containing 1 volume of 0.3% (w/v) *p*-nitrophenyl palmitate in isopropanol and 9 volume of 50 mM Na_2_PO_4_ buffer [0.2% sodium deoxycholate (w/v) and 0.1% gummi arabicum (w/v) (pH 8.0)]. The resultant mixture was incubated for 1 h at room temperature. Following incubation, 1 ml of 1 M sodium carbonate buffer was added to the mixture to terminate the reaction. The resultant mixture was then centrifuged at 10,000 rpm, and the absorbance for the supernatant was read at 410 nm using a spectrophotometer (Srinivasan et al., 2017).

#### Elastase Assay

Elastin Congo Red (ECR; Sigma, St. Louis, MO, United States) was used as a substrate to measure the elastolytic activity of *A. hydrophila*. ECR buffer (900 μl, 100 mM Tris, 1 mM CaCl_2_, pH 7.5) containing 20 mg of ECR was added to 100 μl of filter-sterilized CFCS. The reaction mixture was incubated with shaking at 37°C for 3 h. After incubation, the insoluble ECR was separated by centrifugation at 10,000 rpm, and the supernatant was measured at 495 nm (Ohman et al., 1980).

### *In situ* Microscopic Analysis

For microscopic visualization of *A. hydrophila* biofilm formation, the *A. hydrophila* cells could form biofilm on glass slides (1 × 1 cm) placed in 24 wells MTP supplemented with and without NA (93.75–750 μg/ml), and they were incubated for 24 h at 30°C in static condition. After incubation, the glass slides were washed with distilled water and processed as follows.

For light microscopic visualization, the glass slides were washed with distilled water and stained with a 0.4% CV solution for 3 min. The stained-glass slides were then washed with distilled water, air dried, and then mounted on a microscopic slide with the biofilm directed upwards and imaged using a light microscope (Nikon Eclipse Ti 100, Tokyo, Japan) at a magnification of ×400 (Sivaranjani et al., 2018).

For confocal laser scanning microscopic (CLSM) analysis, the glass slides were washed with distilled water and stained with 0.1% acridine orange solution (w/v) (Sigma, St. Louis, MO, United States) for 1 min. The stained-glass slides were washed with distilled water and air dried at room temperature. Then, the stained slides were imaged using CLSM (Model LSM 710, Carl Zeiss, Germany) at ×200 magnification (Bakkiyaraj and Karutha Pandian, 2010).

### Fourier Transform Infrared (FT-IR) Spectral Analysis

*A. hydrophila* cells cultured in the presence and absence of NA was subjected to FT-IR spectral analysis to disclose the alterations in the cell membrane. An equal volume (1 mg) of lyophilized bacterial cells was mixed with potassium bromide (100 mg, KBr) powder and then ground to prepare bacterial pellets. These pellets were analyzed using an FT-IR spectrophotometer (Nicolet^TM^ iS5, Thermo Scientific, United States). The pellets were scanned at 4,000–400 cm^–1^. Each spectrum represents a total of 16 scans with a spectral resolution of 4 cm^–1^. KBr pellets without bacterial cells were used to reduce the background noise. The spectral readings were plotted as absorbance versus wavenumber and analyzed using OMNIC software (Kannappan et al., 2019b).

### RNA Isolation and cDNA Conversion

The effect of NA at MBIC on the expression of key virulence genes in *A. hydrophila* was quantified. Total RNA was extracted from *A. hydrophila* cells harbored in the presence and absence of NA at MBIC using TRIzol reagent (Sigma, St. Louis, MO, United States). According to the manufacturer’s instructions, extracted RNA was converted to cDNAs using a High-Capacity cDNA Reverse transcription kit (Applied Biosystems).

### Comparative qRT-PCR Analysis

An equal volume of cDNA (2 ng) was combined individually with primers of the target gene (*aerA*, *ahh1*, *lip*, and *ahyB*) and reference gene (16S rRNA) of *A. hydrophila* (Table 1) and Power SYBR^®^ Green PCR master mix (Applied Biosystems). The qRT-PCR was processed with Applied Biosystem’s 7,500 sequence detection system. The PCR cycling conditions include an initial denaturation at 95°C for 5 min, denaturation at 95°C for 40 s, annealing at 57°C for 45 s and extension at 72°C for 40 s (40 cycles) and a final extension at 72°C for 10 min. Data were normalized with the reference gene and analyzed by the 2^–ΔΔCT^ method (Kannappan et al., 2017a).

**TABLE 1:** List of primers used in the gene expression studies.

S. No	Gene	Gene name	Primer sequence (5′–3′)		Size (bp)	Accession numbers	References
			Forward	Reverse			
1	*ahh1*	Hemolysin	GCCGAGCGCCCAGAAGGTGAGTT	GAGCGGCTGGATGCGGTTGT	130	A0A3T-0ZZY5	Wang et al., 2003
2	*aerA*	Aerolysin	CAAGAACAAGTTCAAGTGGCCA	ACGAAGGTGTGGTTCCAGT	309	P09167	Wang et al., 2003
3	*lip*	Lipase	ATCTTCTCCGACTGGTTCGG	CCGTGCCAGGACTGGGTCTT	383	H6VYX6	Sen, 2005
4	16S rRNA	16S ribosomal RNA	ACTCCTACGGGAGGCAGCAG	ATTACCGCGGCTGCTGG	1,400	JN559379	Rama Devi et al., 2016

### Effect of NA on the Growth of *A. hydrophila*

The effect of NA on the growth of *A. hydrophila* was assessed by cell density quantification assay. NA was added to the wells of MTP containing the above said concentrations, ranging from 93.75 to 1,500 μg/ml to 1 ml LB broth, to which 1% of standard cell suspension of *A. hydrophila* culture was inoculated and incubated at 30°C for 24 h. After 24 h of incubation, the cell density was quantified spectrophotometrically at 600 nm (Bakkiyaraj and Karutha Pandian, 2010).

### *In vivo* Analysis

#### Animal Maintenance

Clinically healthy wild type adult zebrafish, *D. rerio* was collected from local ornamental fish farm, Madurai, Tamil Nadu, India. The collected zebrafish were acclimatized at 28 ± 2°C for 7 days in a sterilized glass aquarium and fed with commercial food pellets twice a day. Charcoal-filtered fresh water was used during the study period.

#### Determination of LC_50_

After acclimatization, fish were randomly divided into seven groups (Control group and six experimental groups) with triplicates in 3L glass aquarium tanks (Size—length 240 mm, width 135 mm and height 130 mm) with optimum conditions. Each group contains six healthy fishes of 2.5–3.0 cm length and 3.0–6.0 gm weight. To estimate the lethal concentration 50% (LC_50_), the fishes were exposed to NA at different concentrations (100, 110, 120, 130, 140, and 150 ppm). Control groups were maintained without NA. The concentrations of NA were maintained after every water exchange. Clinical disease symptoms and mortalities were recorded for 96 h. The LC_50_ concentration for 96 h was determined, and the one-tenth value of the LC_50_ concentration was taken as the sublethal concentration for further analysis (Westerfield, 2000).

#### Infectivity Challenge

The immersion route of infection is found to be a suitable experiment method for testing the bacterial virulence. Hence, in this study, the infectivity challenge was carried out in zebrafish according to the immersion challenge test described by Rasch et al. (2004). Zebrafish were immersed in *A. hydrophila* culture at a final concentration of 1 × 10^5^ CFU/ml for 6 h. After immersion, the fish were transferred to their respective aquaria in triplicates. In total, five treatment groups were assigned, and their details are as follows: an uninfected treatment group (control), a post-challenge untreated control group and a post-challenge NA treatment group with three different concentrations (3.5, 7.0, and 14 ppm). Fishes in the uninfected treatment group were not exposed to the test pathogen. Fishes were fed with a normal diet and observed once in every 12 h up to 96 h to record the clinical symptoms and mortalities.

#### Quantification of Bacterial Load

To quantify the bacterial load, individual fish from each treatment group was homogenized and their slurries were made into suspensions. From serially diluted zebrafish slurries, 100 μl was spread onto Aeromonas isolation agar (Commercially purchased from HiMedia India, Catalog code—M884) plates in triplicate and incubated at 30°C for 24 h. Colonies with a distinctive green color were counted to calculate the average number of CFU/ml. Their fold changes were calculated by using the following formula:

Fold⁢change=(CFU/ml⁢of⁢control)/(CFU/ml⁢of⁢treated)

#### Histopathology Analysis

Histopathology is a well-known technique to assess the qualitative changes in the tissue and the patterns of recovery during infection. For histopathological studies, zebrafish tissue specimens, such as gills, muscle, liver, intestine, and kidneys, were taken from uninfected, infected, and NA (14 ppm) treated groups. Tissue specimens were fixed in 10% neutral buffered formalin (NBF) for 24 h and embedded in paraffin wax, and all the tissues were sectioned at 3 μm thickness. All mounted sections were stained with hematoxylin and eosin (H&E) stain (Sheehan and Hrapchak, 1980).

### Statistical Analysis

All the experiments were done in triplicate and repeated at least thrice. Values were expressed as mean ± SD. The statistical analyses were performed using GraphPad Prism v7.0. The Tukey’s multiple comparisons test (one-way analysis of variance) was used to compare the groups for all assays. The Student’s *t*-test was used to compare the control and treated samples for qRT-PCR analysis. All the letters (a, b, c, d, and e) indicate statistically significant at *p* ≤ 0.0001.

## Results

### Determination of Minimum Biofilm Inhibitory Concentration of NA

**FIGURE 1:**
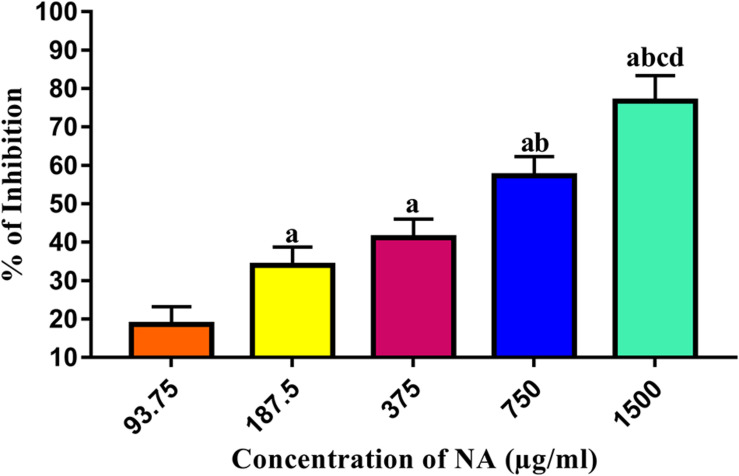
Determination of MBIC of NA against *A. hydrophila* biofilm formation. Results indicate the mean values of three independent experiments and SD. The Tukey’s multiple comparisons test (one-way analysis of variance) was used to compare the groups. ^a^*p* ≤ 0.0001 when compare to control, ^b^*p* ≤ 0.0001 when compare to 93.75 μg/ml, ^c^*p* ≤ 0.0001 when compare to 187.5 μg/ml, and ^d^*p* ≤ 0.0001 when compare to 375 μg/ml.

**FIGURE 2:**
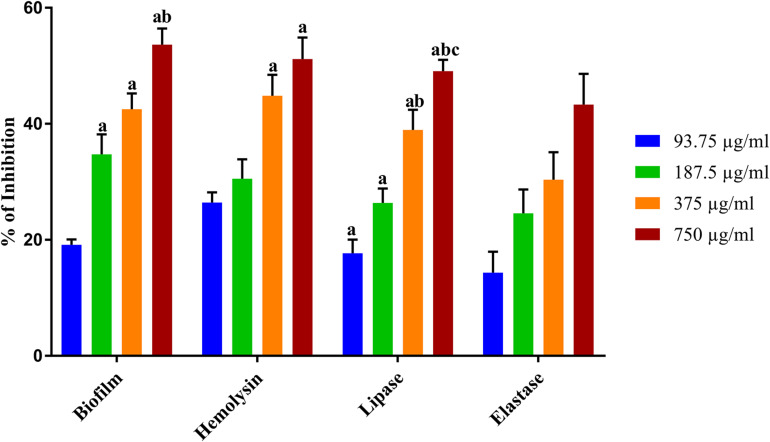
Inhibitory effect of NA on the QS controlled virulence factors production. The graph illustrates percentages of biofilm, hemolysin, lipase, and elastase inhibition in *A. hydrophila* upon treatment with NA at different concentrations (93.75–750 μg/ml). Results indicate the mean values of three independent experiments and SD. The Tukey’s multiple comparisons test (one-way analysis of variance) was used to compare the groups. ^a^*p* ≤ 0.0001 when compare to control, ^b^*p* ≤ 0.0001 when compare to 93.75 μg/ml, and ^c^*p* ≤ 0.0001 when compared to 187.5 μg/ml.

To determine the MBIC, the biofilm biomass inhibition assay was performed with *A. hydrophila* in the presence of NA at doubling concentrations. The results revealed that NA inhibited the biofilm formation of *A. hydrophila* in a concentration-dependent manner (Figures 1, 2). At 750 μg/ml concentration, the NA effectively reduced the *A. hydrophila* biofilm formation up to 55%, and this concentration was considered as MBIC. Therefore, the concentrations ranging from 93.75 to 750 μg/ml were selected for assessing the QS inhibitory potential of NA against *A. hydrophila*.

### QS Inhibition Assay for *A. hydrophila*

#### Effect of NA on β-Hemolysin of *A. hydrophila*

Hemolysin is an exotoxin and the lytic activities on erythrocytes cause anemia in the host. The hemolytic activity in *A. hydrophila* was measured using sheep erythrocytes as substrate. The exposure of *A. hydrophila* to NA at different concentrations (93.75–750 μg/ml) significantly reduced the hemolysin production compared to that of the untreated control (Figure 2). The production of hemolysin was inhibited to 51% upon the treatment with 750 μg/ml concentration of NA.

#### Effect of NA on Lipase of *A. hydrophila*

The lipase is vital for bacterial nutrition and causes damage to the host plasma membrane. A significant decrease in lipase production was found in NA treated *A. hydrophila.* The highest test concentration (750 μg/ml) of NA inhibited a maximum of 49% of lipase production in *A. hydrophila* (Figure 2).

#### Effect of NA on Elastase of *A. hydrophila*

Elastase is one of the extracellular hydrolytic enzymes, which are thought to be a contributor of *A. hydrophila* pathogenesis. In an elastase assay, a concentration-dependent inhibition was observed in elastase production of test pathogen upon treatment with NA (Figure 2). The 750 μg/ml of NA treatment showed a 40% inhibition in *A. hydrophila* elastase production.

### NA Affects Micro-Colony Formation and Biofilm Architecture Development

**FIGURE 3:**
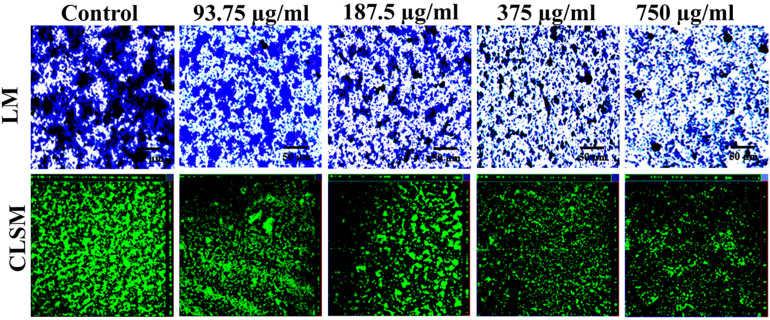
Microscopic validation on the biofilm inhibitory effect of NA against *A. hydrophila*. The light microscopic (LM) and confocal laser scanning microscopic (CLSM) images of *A. hydrophila* biofilm formed in the presence (93.75–750 μg/ml) and the absence (control) of NA.

The microscopic visualization of biofilms demonstrated the effect of NA on *A. hydrophila* biofilm development. The light microscopic examination of *A. hydrophila* harbored NA (93.75–750 μg/ml) on a glass surface showed far less number of micro-colonies compared to the untreated control (Figure 3). The three-dimensional architecture of *A. hydrophila* biofilm in the absence and presence of NA was also studied using CLSM. The confocal images lucidly displayed continuous and well-structured biofilm architecture in untreated control glass slides (Figure 3). In contrast, the *A. hydrophila* slides treated with NA (93.75–750 μg/ml) exhibited disintegration of biofilms along with a significant decrease in the number of micro-colony formation.

### Fourier Transform Infrared (FT-IR) Spectral Analysis

The FT-IR spectral observations exhibited modification in the cellular components of NA treated cells compared to the control cells. In this study, the four most prominent regions corresponding to cellular constituents of NA treated *A. hydrophila* cells were investigated. The predominant regions considered for analysis were 3,500–3,100, 3,000–2,750, 1,800–1,500, and 1,500–1,000 cm^–1^, which corresponds to the hydration, fatty acids, and amide linkage from proteins and peptides and the mixed region, proteins, fatty acids and polysaccharides of bacterial cells, respectively.

**FIGURE 4:**
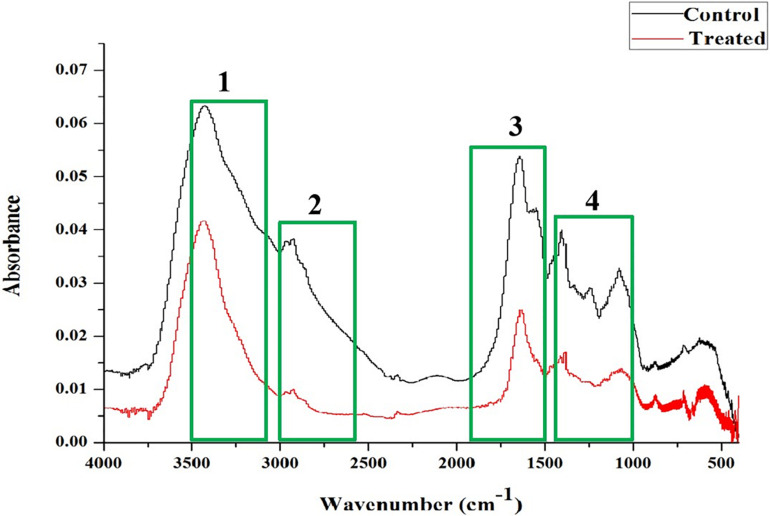
FT-IR spectral analysis showed variation in the absorbance in *A. hydrophila* cells upon treatment with NA (750 μg/ml) compared to the untreated control cells. The regions taken for analysis were shown in the following boxes: (1) 3,500–3,100 cm^–1^ and hydration of microbial cells; (2) 3,000–2,750 cm^–1^ related to fatty acids; (3) 1,800–1,500 cm^–1^: amide linkages within proteins and peptides; and (4) 1,500–1,000 cm^–1^ of a mixed region among proteins and fatty acids of microbial cells.

In FT-IR spectra, NA treated *A. hydrophila* cells showed a declined peak of transmittance in the 3,500–3,100 cm^–1^ region, which indicates enhanced hydration in NA treated *A. hydrophila* cells than the control cells. The vast difference in the transmittance was observed in NA treated bacterial cells at 3,000–2,750 cm^–1^ region. It reflects a massive reduction in fatty acids in treated *A. hydrophila* cells. In 1,800–1,500 cm^–1^ region, NA treated *A. hydrophila* cells showed declension in the transmittance peak when compared to the control cells, which implies a reduction in proteins and peptides. The descending peak of transmittance was observed in treated *A. hydrophila* cells at 1,500–1,000 cm^–1^, which signifies a considerable reduction in the mixed region for protein and fatty acids (Figure 4). The overall outcome of the FT-IR analysis revealed that NA treatment made considerable alterations in the cellular components of *A. hydrophila*.

### Impact of NA on Virulence Gene Expressions

**FIGURE 5:**
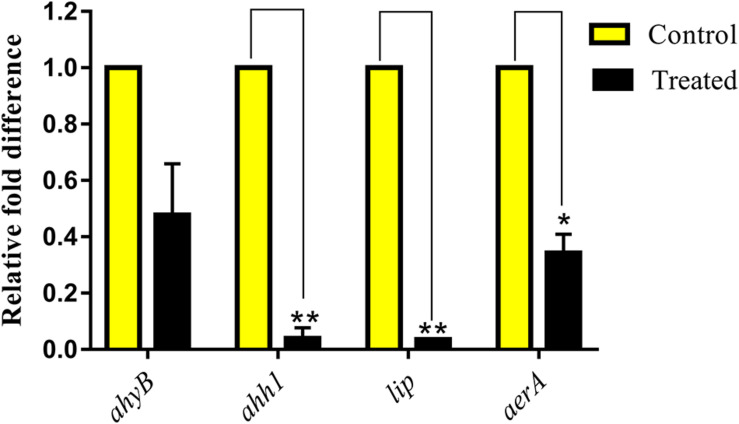
Effect of NA at MBIC on the relative expression of virulence genes in *A. hydrophila*. NA treatment (750 μg/ml) downregulated the QS-controlled virulence genes expression in *A. hydrophila*. Results indicate the mean values of three independent experiments and SD. The Student’s *t*-test was used to compare the control and treated data from qRT-PCR analysis. *p ≤ 0.0308 and **p ≤ 0.0033.

Quantitative real time-PCR (qRT-PCR) has turned out into a precise technique used for gene expression analysis owing to its high specificity and sensitivity. In the present study, the qRT-PCR analysis was carried out to assess the differential gene expression of virulence-associated genes in *A. hydrophila* cells harbored with and without NA. The qRT-PCR results confirmed the downregulation of *ahyB, ahh1*, *lip*, and *aerA* genes up to 0.5-, 0.9-, 0.9-, and 0.6-fold, respectively, upon treatment with NA (Figure 5).

### NA Exhibits Non-bactericidal Activity

**FIGURE 6:**
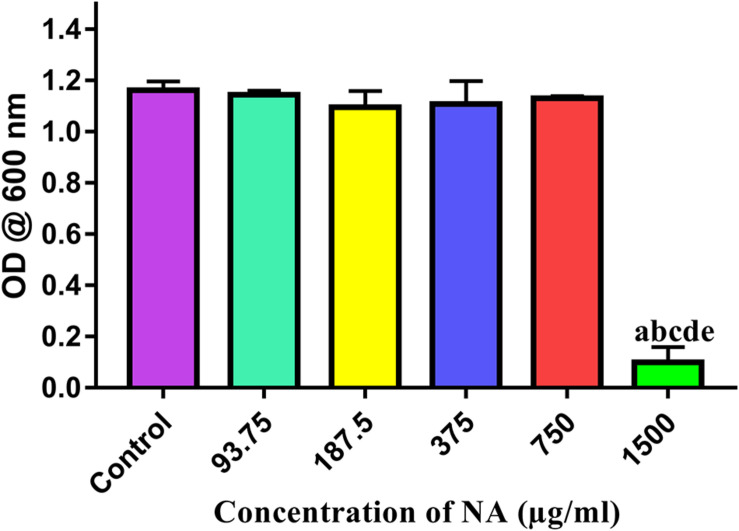
Effect of different concentrations of NA (93.75–1,500 μg/ml) on the growth of *A. hydrophila*. Results indicate the mean values of three independent experiments and SD. The Tukey’s multiple comparisons test (one-way analysis of variance) was used to compare the groups. ^*a*^*p* ≤ 0.0001 when compare to control, ^b^*p* ≤ 0.0001 when compare to 93.75 μg/ml, ^c^*p* ≤ 0.0001 when compare to 187.5 μg/ml, ^d^*p* ≤ 0.0001 when compare to 375 μg/ml, and ^e^*p* ≤ 0.0001 when compare to 750 μg/ml.

Growth of *A. hydrophila* was studied when exposed to NA at different concentrations ranging from 93.75 to 1,500 μg/ml. The spectrophotometric assessment of cell density has revealed no difference in their OD values between the control and the test concentrations of NA up to 750 μg/ml concentration. At the highest concentration (1,500 μg/ml), NA exhibited significant antibacterial activity. This result evidenced the non-bactericidal nature of NA at the QS inhibitory concentration, which is 750 μg/ml (Figure 6).

### *In vivo* Challenge Assay

#### Determination of LC_50_

**FIGURE 7:**
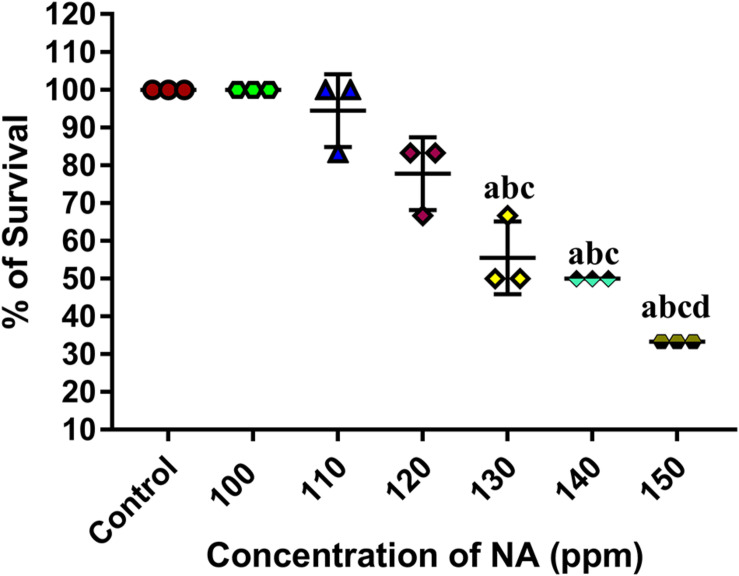
Determination of LC_50_ value of NA on zebrafish. Results indicate the mean values of three independent experiments and SD. The Tukey’s multiple comparisons test (one-way analysis of variance) was used to compare the groups. ^a^*p* ≤ 0.0001 when compare to control, ^b^*p* ≤ 0.0001 when compare to 100 ppm, ^c^*p* ≤ 0.0001 when compare to 110 ppm, and ^d^*p* ≤ 0.0001 when compare to 120 ppm.

At 96 h of incubation, 140 ppm of NA kills half of the total population of zebrafishes. Hence, the same concentration was fixed as the LC_50_ value of NA (Figure 7). One-tenth of the LC_50_ value (14 ppm) was fixed as sub-lethal concentration and the same was used for further anti-infective studies.

#### Effect of NA on Rescuing Post *A. hydrophila* Challenged Zebrafishes

**FIGURE 8:**
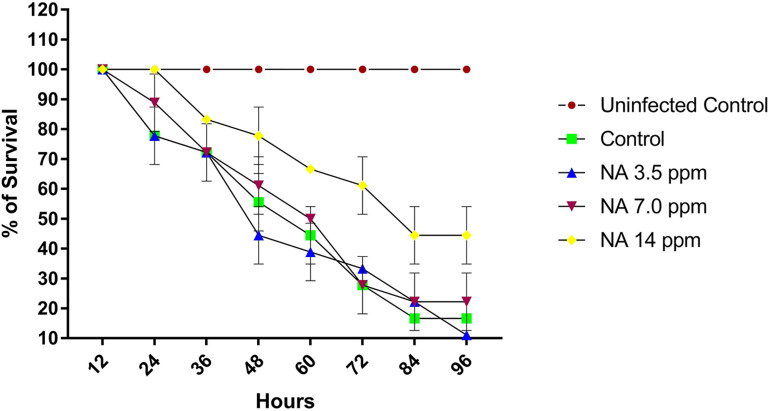
The graph represents the survival percentage of post challenged zebrafishes upon treatment with and without NA at different sub-lethal concentrations (3.5, 7.0, and 14 ppm). Results indicate the mean values of three independent experiments and SD.

The effect of NA on the survival of zebrafishes challenged with *A. hydrophila* was assessed. The uninfected control group showed 100% survival at 96 h. At 24 h of immersion challenge test, zebrafishes started to show clinical signs such as lethargy, increased respiration, abnormal swimming, abdominal ulceration, abdominal dropsy, and bulged eye. At 72 h past the post infection period, the infected control group showed mere survival (28%) of Zebrafishes, whereas the NA treatment (14 ppm) group showed survival of zebrafishes up to 60%. The survival percentage of post challenged zebrafish in the NA treatment group (14 ppm) exhibited 44% at 96 h. In contrast, the 3.5 and 7.0 ppm of NA treatment showed nearly similar level of survival rates to that of the infected group at 96 h (Figure 8).

#### *A. hydrophila* Adhesion to Zebrafish

**FIGURE 9:**
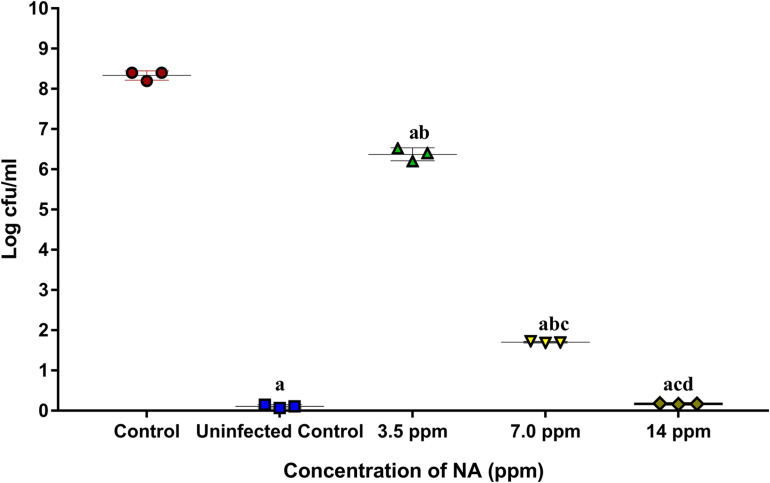
The graph represents CFU counts of *A. hydrophila* in post challenged zebrafish upon treatment with (3.5, 7.0, and 14 ppm) and without NA. Results indicate the mean values of three independent experiments and SD. The Tukey’s multiple comparisons test (one-way analysis of variance) was used to compare the groups. ^a^*p* ≤ 0.0001 when compare to control, ^b^*p* ≤ 0.0001 when compare to uninfected control, ^c^*p* ≤ 0.0001 when compare to 3.5 ppm, and ^d^*p* ≤ 0.0001 when compare to 7.0 ppm.

The surface adhered cells of *A. hydrophila* were recovered from post-challenged zebrafishes to assess the influence of NA in treatment groups. It was found that the adherence of *A. hydrophila* cells on post challenged zebrafishes is much higher than the NA treated zebrafishes. The colony counts accounted from the NA treatment group showed a decreasing unit over the increasing concentration; the fold change was thus increased (Figure 9). The fold changes for 3.5, 7.0, and 14 ppm are 1.3-, 4.8-, and 49-fold in CFU counts, respectively, when compared to the untreated control.

#### Impact of NA on Histopathology of Post-challenged Zebrafish

The present study revealed that the post challenged zebrafish treated with NA manifest morphological alterations in gills, muscle, liver, and intestine and kidney tissue as follows.

##### Histopathology of Gills

**FIGURE 10:**
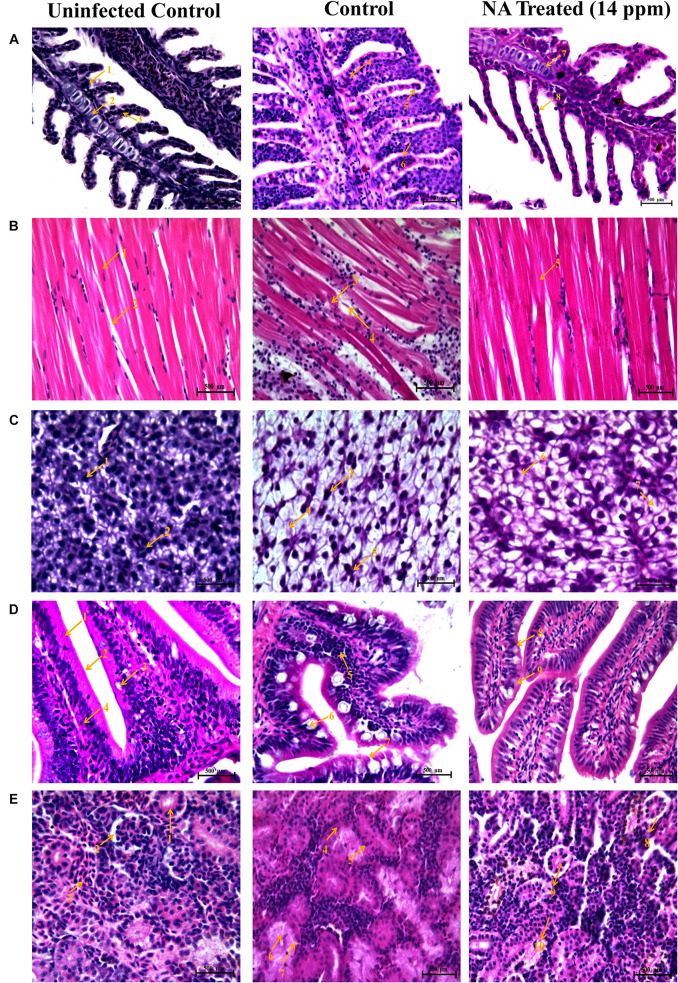
Histopathology analysis of gills **(A)**. 1. Branchial blood vessels; 2. Primary lamella; 3. Secondary lamella; 4. Fusion of secondary lamella; 5. Lamellar lifting; 6. Proliferation of filamentary epithelium; 7. Normal branchial blood vessels; 8. Rescued & healthy secondary lamellae. Histopathology analysis of muscle **(B)**. 1. Muscle bundles; 2. Muscle fiber; 3. Degeneration of muscle bundles; 4. Swelling of muscle fiber; 5. Healthy muscle bundles. Histopathology analysis of liver **(C)**. 1. Hepatocytes; 2. Spherical nucleus; 3. Fatty acid changes in hepatocytes; 4. Cytoplasmic vacuolation; 5. Hepatocytes disruption; 6. Rescued and healthy hepatocytes; 7. Small cytoplasmic vacuolation. Histopathology analysis of intestine **(D)**. 1. Tunica mucosa; 2. External muscle layer; 3. Goblet cell; 4. Lymphocytes; 5. Degeneration of epithelial cells; 6. Hyperplasia of goblet cells; 7. Collapsed tunica mucosa; 8. Normal goblet cell; 9. Rescued and healthy tunica mucosa. Histopathology analysis of kidney **(E)**. 1. Renal glomerulus; 2. Bowman’s space; 3. Normal distal tubules; 4. Congested distal tubules; 5. Hematopoietic necrosis; 6. Degeneration of renal glomerulus; 7. Reduced bowman’s space; 8. Normal bowman’s space; 9. Normal renal glomerulus; 10. Rescued and healthy distal tubulus.

Histological observations on gills of uninfected control zebrafish showed the typical architecture of gills filaments such as the primary lamellae and secondary lamellae with a scattering of mucus cells on both sides. The gills of post challenged untreated control zebrafish showed the most pronounced histopathological alterations such as lamellar lifting, fusion of secondary lamellar, excessive secretion of mucus on the surface of filaments, and proliferation of filamentary epithelium. However, the post-challenged NA treatment groups did not show any deformities in the gills structure. Gills of post challenged zebrafish treated with NA at 14 ppm concentration supported the typical architecture, as seen in the uninfected control fish (Figure 10A).

##### Histopathology of Muscle

The muscle of the uninfected control zebrafish showed normal arrangements of muscle fibers and muscle bundles. Degeneration and fragmentation of muscle fibers were spotted in the muscle tissue of post challenged zebrafish in the untreated control. However, the NA (14 ppm) treatment restored the normal architecture muscle tissue in post challenged zebrafish, which is similar to the histoarchitecture of muscle from the zebrafish in the uninfected control (Figure 10B).

##### Histopathology of Liver

Histopathological section of the liver in the uninfected control zebrafish exhibited a typical structural organization of hepatocytes with a homogenous cytoplasm and a large spherical nucleus. The liver obtained from post-challenged untreated control zebrafish showed severe fatty acid changes of hepatocytes, a focal area of necrosis, cytoplasmic vacuolation, and hepatocyte disruption. In the present investigation, the liver of NA (14 ppm) treated zebrafish post challenged with *A. hydrophila* showed less damage compared to the zebrafish in the untreated control group (Figure 10C).

##### Histopathology of Intestine

Histological sectioning of intestine from uninfected control zebrafish showed no changes in the intestine architecture. In contrast, histopathological sectioning of post challenged untreated control zebrafish showed anomalies such as an increased number of goblet cells (Hyperplasia), collapsed tunica mucosa and degeneration of epithelial cells. Unlike the uninfected control group, NA treatment restored those deformities rendered by *A. hydrophila* infection in the intestine region (Figure 10D).

##### Histopathology of Kidney

Upon NA treatment, zebrafish challenged with *A. hydrophila* showed hyaline droplets in a few renal tubules. In contrast, the post challenged zebrafish in the infection control group showed degeneration of renal tubule with the formation of hyaline droplets, degeneration of renal glomerulus, and renal hematopoietic necrosis to a very severe degree. Histological sectioning of the uninfected control zebrafish kidney showed the typical structural organization of the nephritic tubules with a well-organized glomerulus (Figure 10E).

## Discussion

QS is a gene regulation system dependent on cell density; it coordinates the expression of various virulence factors and biofilm formation in most of the pathogenic microorganism. *A. hydrophila* is an opportunistic aquatic pathogen responsible for causing frequent outbreaks, and it also has an impact on humans while ingesting contaminated seafood (Rama Devi et al., 2016). Recent studies are also continuously reporting that *A. hydrophila* too regulates the virulence factors production and biofilm formation through QS system (Kozlova et al., 2008). In addition to the presence of antibiotic resistance genes, biofilm-mediated antibiotic resistance induces the severity of *A. hydrophila* infections. To control the bacterial pathogenesis and subsequent infection, targeting QS mechanism has become one of the attractive and best alternative methods.

Several studies are continuously reporting the QS inhibitory potential of phenolic plant extracts, clove oil, and natural compounds against *A. hydrophila* (Husain et al., 2013; Ponnusamy et al., 2013; Rodrigues et al., 2016). However, the necessities for developing new classes of effective QS inhibitors against infectious *A. hydrophila* strains are still in demand. Hence, the present study aimed to delineate the anti-QS and anti-infective potential of NA against *A. hydrophila*. Naringin, a non-toxic, polyphenolic compound, has a myriad pharmacological properties such as anti-oxidant, anti-diabetic, and anti-dyslipidemic effects (Jung et al., 2003; Murunga et al., 2016). Further, several studies have reported the antibacterial, antifungal, and QS-inhibitory potential of various plants and fruits extract containing NA as one of its ingredients (Negi and Jayaprakasha, 2001; Celiz et al., 2011; Hendra et al., 2011; Truchado et al., 2012). In contrast, only a very few studies have investigated the QS inhibitory potential of NA. Annapoorani et al. (2012) have reported NA as an effective inhibitor against *Serratia marcescens* QS mechanism. However, to the best of our knowledge, NA has not been previously reported for its anti-QS property against *A. hydrophila*. Hence, an attempt was taken in the present study to explore the anti-QS and anti-infective efficacy of NA against infectious *A. hydrophila*.

Targeting the QS-dependent biofilm formation is an effective alternative treatment strategy to control bacterial infections (Ramanathan et al., 2018). In this study, the obtained result from the biofilm inhibition assay revealed that NA inhibits biofilm formation of *A. hydrophila* in a concentration-dependent manner (Figures 1, 2). Further, the 750 μg/ml concentration of NA was determined as MBIC up to these concentrations as thus taken for further assays. In line with the result, vanillin, a naturally occurring organic compound, has been reported for its anti-biofilm potential against *A. hydrophila* biofilm formation (Ponnusamy et al., 2013). The QS regulatory mechanisms of *A. hydrophila* are closely linked to biofilm formation along with the production of several virulence factors. *A. hydrophila* expresses diverse virulence enzymes production that contribute to its pathogenicity (Igbinosa et al., 2012). Hemolysins are one such group of multifunctional enzymes that involve pore formation in the target cell membrane (Asao et al., 1984). Lipase has been found to damage the plasma membrane of the host cells, and elastase secreted by *A. hydrophila* causes diseases in fish and humans (Stehr et al., 2003; Nam and Joh, 2007). The reduction in the virulence factors production directly evidenced the anti-QS potential of compounds against pathogenic bacteria. Compared to the normal circumstances, *A. hydrophila* harbored in the presence of NA at MBIC was shown to have a decreased extracellular virulence enzymes production level (Figure 2). This result falls in line with the previous findings, wherein the rosmarinic acid at its tested concentration effectively inhibited the production of QS controlled virulence factors such as hemolysin, lipase and elastase and biofilm formation in a pathogenic strains of *A. hydrophila* (Rama Devi et al., 2016).

Schuster and Markx (2013) have stated that the nature and architecture of biofilm prevent the penetration of antibiotics and inhibit their contact with bacterial cells. The anti-biofilm activity of NA was studied by the light microscopic and CLSM analyses. In this study, NA has inhibited the biofilm development of *A. hydrophila* and subsequently reduced the surface area coverage of biofilm. Unlike untreated control images, the images of the treatment group displayed a massive reduction in biofilm architecture development and micro-colony formation in *A. hydrophila* (Figure 3). The results of the light microscopic and CLSM analyses are in agreement with the findings of Alexpandi et al. (2019), wherein *Diplocyclos palmatus* methanolic leaf extract treated *S. marcescens* displayed the collapsed biofilm architecture with a reduction in the number of micro-colonies, compared to their respective untreated controls. This perspective of NA brings it one step closer to being an ideal QS inhibitor.

FT-IR is one of the most used techniques in molecular biology for the detection of alterations in the functional groups present in the cellular components, such as nucleic acids, proteins, lipids, and carbohydrates. Hence, the present study utilized the FT-IR technique to probe the variation in cellular components of *A. hydrophila* when treated with and without NA. The FT-IR spectra of NA treatment displayed the variations in the absorbance peaks correspond to the hydration of bacterial cells, reduction in the level of lipids, proteins, peptides, and polysaccharides compared to the control (Figure 4). Thus, the reduction in cell-bound peptides and polysaccharides directly substantiates the anti-biofilm potential of NA against *A. hydrophila*. Further, FT-IR spectral observations from the work of Kannappan et al. (2017b) is very much similar to the result observed in the present study, wherein the authors assessed the anti-biofilm activity of geraniol against *Staphylococcus epidermidis* RP62A (Kannappan et al., 2017b). Moreover, in this study, the anti-biofilm and anti-QS potential of NA were also assessed at the transcriptional level. The differential expressions of virulence-associated genes were quantified using qRT-PCR analysis. Among the genes used in the study, *ahh1 and aerA* are hemolytic toxin genes responsible for pore formation in host cells, which leads to apoptosis (Heuzenroeder et al., 1999; Wang et al., 2003). These two-component hemolytic systems (*ahh1 and aerA*) are considered to be the prime regulators for the expression of the Aeromonas virulence genes. Further, Wong et al. (1998) have stated that these two-component hemolytic systems should need to abolish for attenuating the virulence of *A. hydrophila* (Wong et al., 1998). The obtained expression data showed a perceptible downregulation of both the *ahh1* and *aerA* genes upon NA treatment (Figure 5). Therefore, it confirms that the NA treatment attenuated the major virulence of *A. hydrophila*. Moreover, the qRT-PCR analysis disclosed the downregulation of *lip* and *ahyB* genes upon treatment with NA (Figure 5). The downregulation of *lip* gene expression in this study goes in parallel with the earlier report of Rama Devi et al. (2016) where the rosmarinic acid downregulated the expression of the lipase gene in *A. hydrophila*. Overall, the gene expression analysis validated the outcome of physiological assays and elucidated the anti-QS potential of NA. It has also been well stated that ideal QS inhibitors should not interfere with the growth of bacteria. To evidence the statement, the concentrations used in the MBIC assay were assessed for their growth inhibitory activity using cell density quantification assay. Up to a 750 μg/ml concentration, NA did not show any effect on the growth of *A. hydrophila*. At 1,500 μg/ml, NA completely suppress the *A. hydrophila* growth (Figure 6). It indicated that the NA at the tested concentration targets only the QS mediated gene expressions of *A. hydrophila* and not its growth. This result therefore publicized that NA is a potential QS inhibitor with no influence on the growth of *A. hydrophila*.

The outcomes of *in vitro* studies have suggested that NA inhibits the QS mediated biofilm and virulence factors production in *A. hydrophila*. To further manifest this, *in vivo* experiments were carried out using zebrafish (*D. rerio*) as a model organism. Zebrafish is a freshwater fish; it has been used for laboratory purposes due to its small size, short life cycle and high fecundity. In this study, the NA was applied in the aquaria water to treat *A. hydrophila* infection in Zebrafish. To determine the LC_50_ value of NA, the survival assay was performed with the different concentrations of NA (100–150 ppm). The obtained data displayed that the LC_50_ value of NA was 140 ppm (Figure 7). One-tenth of the LC_50_ value was thus taken as a sublethal concentration of NA for *in vivo* analysis. The *in vivo* colonization ability of *A. hydrophila* aids the successful establishment of infection in the host (Natrah et al., 2012). *A. hydrophila* infection in zebrafish was experimentally induced by immersing the fish in a suspension of the test bacterial pathogen. The result of *in vivo* infectivity assay showed that the NA reduced the infection rate and enhanced the survival of zebrafishes challenged with *A. hydrophila*, compared to the untreated control (Figure 8). The outcome of this result has clearly indicated that the NA treatment significantly enhanced the survival of zebrafishes from *A. hydrophila* infections. Further, Harikrishnan et al. (2009) have observed a similar enhanced survival rate of goldfish (*Carassius auratus*) challenged with *A. hydrophila* upon treatment with triherbal solvent extracts. Furthermore, the CFU counting assay validated the outcome of survival assay, in which the NA treatment showed a decreased level of CFU counts in zebrafish, compared to the untreated control (Figure 9). The outcome of these results is going parallel with the findings of Pachanawan et al. (2008), who studied the protective effect of flavonoids from ethanolic leaf extract of *Psidium guajava* against *A. hydrophila* in tilapia fish.

To confirm the anti-infective efficacy of NA, the histopathological analysis was documented for NA treated and untreated vital organs (gills, muscle, liver, intestine, and kidney) of zebrafish. Gills of fish are predominantly sensitive to chemical and physical changes in the aquatic environment. The liver can detoxify toxic compounds. The presence of toxic compounds at high concentrations cause structural damages in the liver cells (Bruslé and Anadon, 1996; Camargo and Martinez, 2007). The intestine of a fish is the primary site for food digestion and nutrient absorption. Furthermore, the liver and kidney are easily infected by contaminants in water (Thophon et al., 2003). In this study, the *A. hydrophila* infected fish showed acute hemorrhage and necrosis in vital organs such as the liver, intestine, and kidney due to the production of toxins and extracellular enzymes by *A. hydrophila*. This data have supported the outcome of Gado (1998) and Afifi et al. (2000), who have reported that the extracellular virulence enzymes and toxins produced by *A. hydrophila* leads to rapid death in fishes as a result of organ failure. Furthermore, the histological observations of vital organs from the zebrafish of uninfected control showed typical histoarchitecture. In contrast, the histological sectioning of post-challenged zebrafish from control group showed significant histological alterations by *A. hydrophila* infection in all vital organs such as fusion and increased thickness of secondary lamellar in gills, the structural damages observed in muscle sectioning, vacuolation in liver hepatic cells., increased goblet cell secretion and epithelial cell necrosis in intestine and tubule degeneration and hematopoietic tissue necrosis in kidney. A similar kind of annotations was previously observed by Gobinath and Ramanibai (2014), who have reported the adverse histological variations in different vital organs of *Labeo rohita* such as the gills, muscle, liver, and kidney upon *Vibrio cholerae* infection. In contrast to the untreated control, the NA treatment did not show any adverse pathological changes in vital organs of zebrafish. Moreover, the normal structural architecture was observed in NA treatment as like uninfected control (Figure 10). Therefore, the findings of this *in vivo* study revealed that the NA treatment increases the defending ability of zebrafish against *A. hydrophila* infection and in turn reduces the disease susceptibility.

In conclusion, for the first time, NA has proven to be a potential anti-QS and anti-infective agent against *A. hydrophila*. The results of microscopic, spectroscopic, and differential gene expression analyses have substantiated our perception over NA as an anti-QS agent. Furthermore, the *in vivo* experiments with zebrafish as model system have confirmed the anti-infective potential of NA. Overall, this study clearly reveals the *in vitro* and *in vivo* therapeutic potential of NA against *A. hydrophila* infection. Furthermore, clinical studies evaluating the potential of NA against *A. hydrophila* infection and elucidating the molecular mechanism behind anti-QS activity of NA, which could pave the way to develop NA as a potential drug candidate against *A. hydrophila* pathogenesis.

## Data Availability Statement

The original contributions presented in the study are included in the article/supplementary material, further inquiries can be directed to the corresponding author/s.

## Ethics Statement

All the zebrafish experiments in this study were done in accordance with the guidelines of Committee for the Purpose of Control and Supervision of Experiments on Animal (CPCSEA) (cpcsea.nic.in/WriteReadData/userfiles/file/SOP_CPCSEA_inner _page.pdf), Government of India and general guidelines of Institutional Animal Ethics Committee, Alagappa University. Therefore, ethical approval is not required for the animal work in this study.

## Author Contributions

RS, KD, AR, and XL conceptualized and validated the study. RS and KD performed the methodology and the data curation, and wrote and prepared the manuscript for the final draft. RS and AK performed the formal analysis. RS, KD, SS, and AK investigated the study. AR and XL were responsible for the resources, supervised the study, and performed the funding acquisition. RS, AK, AR, and XL visualized the study. RS, KD, and AK wrote, reviewed, and edited the manuscript. All authors contributed to the article and approved the submitted version.

## Conflict of Interest

The authors declare that the research was conducted in the absence of any commercial or financial relationships that could be construed as a potential conflict of interest.
